# Faculty development for strengthening online teaching capability: a mixed-methods study of what staff want, evaluated with Kirkpatrick’s model of teaching effectiveness

**DOI:** 10.12688/mep.19692.2

**Published:** 2023-11-30

**Authors:** Rachelle Singleton, Daniela Ruiz Cosignani, Monica Kam, Megan Clune, Amanda Charlton, Tanisha Jowsey

**Affiliations:** 1 School of Medical Sciences, The University of Auckland, Faculty of Medical and Health Sciences, Auckland, New Zealand; 2 Centre for Medical and Health Sciences Education, The University of Auckland, Faculty of Medical and Health Sciences, Auckland, New Zealand; 3 School of Biological Sciences, The University of Auckland, Auckland, New Zealand; 4 School of Curriculum and Pedagogy, The University of Auckland, Faculty of Education and Social Work, Auckland, New Zealand; 5 Department of Anatomical Pathology, Auckland City Hospital, Auckland, New Zealand; 6 Department of Molecular Medicine and Pathology, The University of Auckland, Faculty of Medical and Health Sciences, Auckland, New Zealand

**Keywords:** Online teaching, faculty development, professional development, interactive learning, H5P

## Abstract

**Background:**

Globally, tertiary teachers are increasingly being pushed and pulled into online teaching. While most developments in online education have focused on the student perspective, few studies have reported faculty development (FD) initiatives for increasing online teaching capability and confidence from a staff perspective.

**Methods:**

We designed and evaluated FD workshops, using five datasets, and the use of H5P software for interactive online teaching. We used educational theory to design our FD (Mayer multimedia principles, active learning) and evaluated our FD initiatives using the Best Evidence Medical Education (BEME) 2006 modified Kirkpatrick levels.

**Results:**

Teaching staff reported that Communities of Practice were important for their learning and emotional support. Uptake and deployment of FD skills depended on the interactivity of FD sessions, their timeliness, and sufficient time allocated to attend and implement. Staff who applied FD learning to their online teaching created interactive learning resources. This content was associated with an increase in student grades, and the roll-out of an institutional site-wide H5P license.

**Conclusion:**

This paper demonstrates an effective strategy for upskilling and upscaling faculty development. The use of H5P as a teaching tool enhances student learning. For successful FD, we make four recommendations. These are: provide just-in-time learning and allocate time for FD and staff to create online teaching material; foster supportive communities; offer personalized support; and design hands on active learning.

## Introduction

With the rapid pivot to online tertiary education, many teaching staff have struggled to adapt from on-campus to online teaching due to a lack of capability and confidence. A recent Best Evidence Medical Education (BEME) scoping review ( Daniel *et al.*, 2021) highlighted a surprising lack of research on faculty development (FD) initiatives from the teaching staff perspective. Understanding what staff want to teach effectively online is necessary for effective FD initiatives. Effective and sustainable FD initiatives are urgently required to meet the growing demand for online education.

In this paper we draw on educational theory to design our FD (Mayer multimedia principles Mayer (2017), active learning) and evaluate our FD initiatives using the BEME 2006 modified Kirkpatrick levels ( Steinert *et al.*, 2006), see *Extended data* ( Singleton *et al*., 2023) As per best practice with curricula and programmatic evaluation, we explore perspectives from both teaching staff and students.

We use Steinert *et al*.'s (2016) definition of FD as any formal or informal activity carried out by teaching staff to improve their knowledge, skills, and behaviors as teachers, leaders, managers, researchers, or scholars. For this study, we define 'teaching staff' as staff who are responsible for students' learning, including support staff who assist students, such as learning advisors, librarians, graduate teaching assistants and staff who contribute to teaching administration.

### Research questions

1. What do teaching staff want from FD initiatives to strengthen their online teaching?
2. Can we demonstrate teaching effectiveness after incorporating our FD initiatives, evaluated by the BEME 2006 modified Kirkpatrick model?

## Methods

### Ethics

The University of Auckland’s Human Participants Ethics Committee approved this mixed methods study on 25 ^th^ November 2021 (approval number: UAHPEC 23149). Written informed consent was obtained from all participants and all data was de-identified to ensure anonymity.

Research occurred in the Faculty of Medical and Health Sciences (FMHS) at The University of Auckland (UoA), New Zealand. FMHS offers undergraduate and postgraduate courses for many medical and clinical degrees. We surveyed and interviewed UoA staff and ran and evaluated FD initiatives from March 2020 to March 2022. Using educational theory and design thinking, we created FD workshops to train staff on how to use new educational software ( H5P) for teaching online. To evaluate the educational effect, we collected UoA undergraduate student data from March 2018 to March 2021.

### Datasets

**Figure 1.**
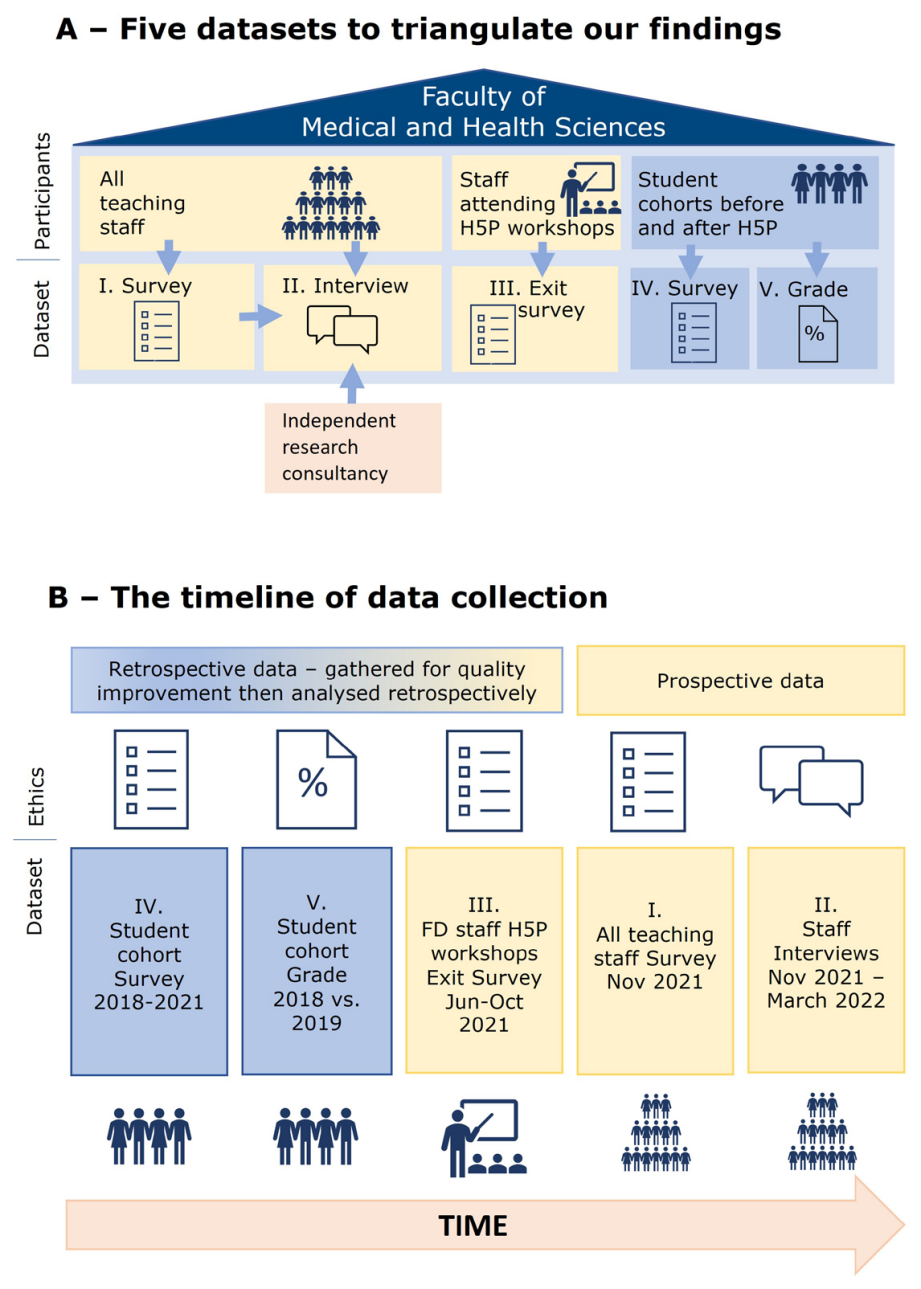
Five datasets to triangulate our findings. **A** – Five datasets to triangulate our findings. **B** – The timeline of data collection. Icons in this figure are Microsoft PowerPoint icons. All authors have a current paid subscription to Microsoft PowerPoint.

We gathered five datasets, which comprised an all-teaching staff survey (staff n=105), interviews (staff n=20), staff H5P workshop exit surveys (staff n=86), student grades and student evaluations (n=809). Figure 1 shows A) how we used the five data sets to triangulate our findings; and B) the timeline of data collection for our study. The datasets are ordered within this paper to prioritise the staff perception of faculty development for teaching. We collected demographic data for all-teaching staff survey and staff interviews. Our primary focus was the teaching staff's perceptions of FD initiatives, therefore the analysis concerned aggregated data.

**Figure 2.**
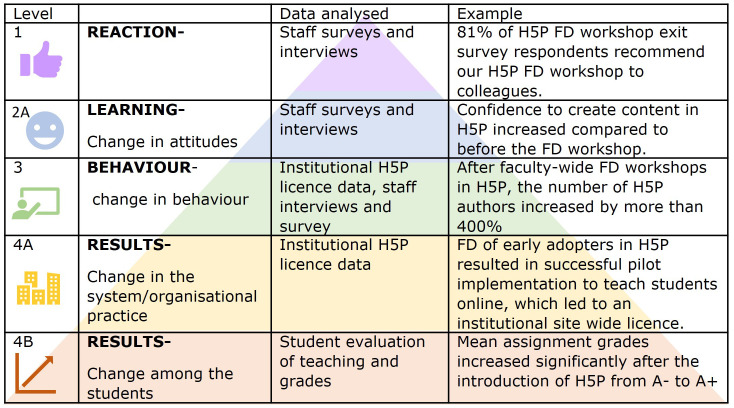
BEME 2006 adaptation ( Steinert *et al.*, 2006) of Kirkpatrick's model for evaluating educational outcomes. Examples illustrate outcomes following Faculty Development (FD) workshops to use H5P. Icons in this figure are Microsoft PowerPoint icons. All authors have a current paid subscription to Microsoft PowerPoint.

Each dataset speaks to different levels of evaluation using the BEME 2006 adaptation ( Steinert *et al.*, 2006) of Kirkpatrick’s Four-Level Model (BEME evaluation) to evaluate the teaching effectiveness of FD initiatives (see *Extended data* ( Singleton *et al*., 2023)). Datasets I and II capture “what staff want” in terms of increasing their confidence and capability to teach online. Dataset III captures the reaction and learning of teaching staff after their H5P workshop (BEME evaluation levels 1 and 2A). Datasets IV and V provide results of implementing H5P (BEME evaluation level 4B) with students. We used institutional H5P license usage data to evaluate staff behavior change and adoption of H5P use (BEME evaluation level 3 and 4B). Figure 2 shows the description, data analyzed and example from our results at each BEME evaluation level.

### Data collection: sampling and saturation

*Dataset I:* We designed the all-teaching staff survey (dataset I) (see *Extended data* ( Singleton *et al*., 2023)) and interviews (dataset II) (see ESM C) to capture a holistic view of the teaching staff’s perceptions of all the FD available, including our H5P workshops (dataset III) and how they have applied any knowledge or skills acquired from them. Author MC entered the survey questions into *Qualtrics* . Authors RS, DRC, MK and TJ then completed the survey and refined it. Author RS emailed in November 2021 a link to the *Qualtrics* survey to an existing FMHS teaching and learning community email distribution list with approximately 460 members. Eligible participants were academic and professional staff within the FMHS who were in a teaching role or contributed to teaching or learning design from March 2020 to December 2021. “Teaching role” is defined as educators who were responsible for students’ learning within the FMHS. This includes course teaching but also includes support staff who assist students in learning contexts such as learning advisors, librarians, graduate teaching assistants, teaching assistants and professional staff who contribute to teaching administration (e.g., Canvas). We included demographic questions such as gender and ethnicity in this survey (Dataset I). Following face validity, we undertook pilot testing of dataset I (all-teaching staff survey) with the first five survey responses, minor changes were made and the recruitment continued. As this was a descriptive study, we did not undertake further validity and reliability testing of the dataset I survey. We closed the all-teaching staff survey (Dataset I) at 86 respondents when no new responses were received after three email reminders.

The survey included 38 questions, a mix of open-ended text box responses and selected choice answers. Author RS led descriptive analysis of selected choice responses using Microsoft Excel®. Author AC led the thematic analysis of open-ended questions. We followed Braun and Clarke’s (2012) practical six-phase approach: 1) Familiarizing researchers with data, 2) Generating initial codes, 3) Searching for themes, 4) Reviewing themes, 5) Defining and naming themes, and 6) Producing the report. The whole research team (RS, DRC, MK, MC, AC and TJ) met regularly throughout this process to discuss any analytical issues arising and reach a consensus before progressing the analysis.

*Dataset II:* We included the invitation to an interview (dataset II) by an external link at the end of the Qualtrics all-teaching staff survey (dataset I). Consent to participate in the interview component was sought via an electronic sign-up sheet (Qualtrics) that required ticking three boxes to ensure requirement were met as per the participant information sheet. The electronic sign-up sheet also asked for an electronic signature as final confirmation of consent. The two Qualtrics forms (all-teaching staff survey and interview sign-up sheet) were not linked, so anonymous survey data could not be identified by signing up to an interview. The names and email contact details of people who volunteered to participate in an interview were provided to an external consulting agency, Academic Consulting LTD. We outsourced collection of the interview data to this agency in order to minimize bias in the study. Academic Consulting LTD signed a confidentiality agreement.

Qualitative methods sampling continues until data saturation—when no new information emerges from the collected data—which, according to Ando *et al.* (2014), often occurs with 12 participants in a relatively homogenous sample. We continued beyond 12 until we were sure that data saturation had been reached. We stopped interview recruitment at 20 participants.

Authors RS, TJ and AC went through an iterative approach to refining the interview question guide (see *Underlying data* ( Singleton *et al*., 2023)) with Academic Consulting LTD. Interviews took place between November 2021 and March 2022 and were audio-recorded and transcribed verbatim via Zoom. Academic Consulting LTD de-identified interview data and led the thematic analysis using QSR Nvivo Software (propriety software) to support analysis. Several similar free qualitative software programs exist (such as RQDA) and analysis can also be undertaken manually. Thematic analysis of the interview transcripts followed Braun and Clarke’s (2012) practical six-phase approach. Following analysis, Academic Consulting LTD sent the authors (RS and TJ) the de-identified Nvivo database (see *Underlying data* ( Singleton *et al*., 2023)) and findings report, after which they deleted their copy of the data.

*Dataset III:* The H5P workshop exit survey (see *Extended data* ( Singleton *et al*., 2023) ESM D) incorporated elements of two existing surveys, Brookfield’s (1998) critical incident questionnaire; captured participants’ reactions in the emotional domain using an emoji sentiment scale ( Marder *et al.*, 2020), and used the Net Promoter Score ( Reichheld, 2003) as a global satisfaction metric. Authors AC and MC created this survey in *Qualtrics* and designed the survey to inform workshop improvement. Authors RS, TJ, MK and DRC completed the survey and refined it. As this is a descriptive study, we did not undertake further validity and reliability testing of the dataset III survey. Authors RS and AC distributed these surveys at the end of each H5P FD workshop and obtained an approximate 30% response rate. Workshop exit surveys informed iterative design of subsequent workshops within a series of six workshops held across June - October 2021. Completion of the H5P workshop exit survey was voluntary and anonymous. Workshop participants were academic and professional staff within the FMHS who were in a teaching role or contributed to teaching or learning design from March 2020 to December 2021. When we ran the workshops we designed and administered the survey with quality improvement – not research – in mind.

The survey included a mix of open-ended text box responses and selected choice answers. Descriptive analysis of selected choice responses was performed by RS using Microsoft Excel®. Author AC led the thematic analysis of open-ended questions, following Braun and Clarke’s (2012) practical six-phase approach. The whole research team (RS, DRC, MK, MC, AC and TJ) met regularly throughout this process to discuss any analytical issues arising and reach a consensus before progressing the analysis.

*Dataset IV:* Author RS collected routine anonymous Student Evaluation of Teaching [SET] and formative evaluations from the course ‘MEDSCI 203 – Mechanisms of Disease’ from 2018 to 2021. Students were invited to participate in SET in the last week of teaching each semester, via Learning Management Software (LMS) notifications. Students were asked about what helps them learn and what can be improved in a course (see *Underlying data* ( Singleton *et al*., 2023)). The student evaluations included a mix of open-ended text box responses and selected choice answers. Authors RS and MK independently analyzed the open-ended question data using thematic analysis following Braun and Clark’s (2012) practical six-phase approach, for student perceptions of the course before and after incorporating interactive online learning resources, first introduced in 2019 by author RS, an early adopter of H5P. An example of a technology-enhanced interactive learning activity created with H5P software is provided. Readers can experience this activity here. See also *Extended data* ( Singleton *et al*., 2023). This activity requires students to read scientific articles selected to complement material taught in lectures, and then complete a variety of online interactive activities aimed to help ease them into reading and appraising scientific literature. The embedded interactive online learning activities in this assignment exemplify many of the recommendations of active learning with personalized feedback.

The student evaluation response rate range was 14.1 – 97% out of a class of 182 (2018) – 314 (2021) students per year. The research team met regularly throughout the thematic analysis process to discuss any analytical issues arising and reach a consensus between the themes identified by RS and MK, before progressing the analysis.

*Dataset V:* Student assignment grades were captured from the LMS for the course ‘MEDSCI 203 – Mechanisms of Disease’ from 2018 to 2019. RS and MC then analyzed the student grades quantitatively using descriptive analyses and t-test analyses in Microsoft Excel® to determine the significance ( *p*<.001) of changes in assignment grades between 2018 and 2019, before and after the introduction of interactive online H5P resources in 2019.

### Bias, positionality, and reflexivity statements

Jowsey *et al*. (2021) writes, ‘Bias is an inclination or prejudice for or against someone or something, whereas positionality is a person’s position in society and/or their stance towards someone or something’. At the time of data collection authors RS, MK, MC, TJ were members of the teaching and academic staff of the UoA. Author DRC was a student of the UoA (Master of Clinical Education) and a specialist Endodontist. Author AC is a Pathologist and was adjunct academic of the UoA. We were all early adopters of H5P, facilitated staff workshops about H5P, and are biased towards it. Therefore, we outsourced the staff interview data collection and analysis to an external consulting agency, Academic Consulting LTD.

### FD workshop design

The theoretical underpinnings of our FD workshop design are as follows:

*Multimedia learning:* Mayer (2017) developed an evidence-based cognitive theory of multimedia learning on how to structure multimedia effectively to maximize learning. We incorporated Mayer’s principles to design our FD workshops, particularly multimedia, signaling, segmenting, modality, and personalization.

*Intrinsic motivation and Self Determination Theory:* Learner intrinsic motivation is associated with greater engagement, deep learning, and higher academic performance than extrinsic motivation. Self-determination theory requires the three basic psychological needs of autonomy, competence, and relatedness must be fulfilled to support and sustain intrinsic motivation ( Deci & Ryan, 1985). We designed FD initiatives to support all three needs with particular attention to relatedness by incorporating socio-communicative elements ( Blumenstein, 2020), which are analyzed by indicators of online social engagement ( Redmond *et al.*, 2018).

*Personalized learning:* Redecker *et al.* (2010) recommend that professional development encompass personalization, including tailor-made and targeted learning pathways that are motivating and engaging, but also, efficient and relevant. In a meta-analysis of online learning design, Blumenstein (2020) identified learners valued tailored feedback and individualized messages, and recommended these are incorporated into a more holistic perspective, including wellbeing and emotional support.

*Diffusion of innovation theory*: Kaminski (2011) writes that the Diffusion of Innovation Theory ( Rogers *et al.*, 2014) ‘is often regarded as a valuable change model for guiding technological innovation where the innovation itself is modified and presented in ways that meet the needs across all levels of adopters’. In our case of online learning design as innovation, the turn of the 21 ^st^ century saw innovators and early adopters entering the innovation curve, which accelerated rapidly with the COVID-19 pandemic ( Jowsey *et al*., 2020). Our FD initiatives were timed to support the Diffusion of Innovation for majority adoption.

We used principles from the iterative design thinking process ( Brown & Katz, 2011) to construct our workshop for FD. The following steps loosely guided the design process: empathize, define, ideate, prototype, and test ( Plattner *et al.*, 2010). We informally identified what staff wanted, then defined the problems to be addressed, created a workshop that we believed would address these, and delivered the workshop. Then we iteratively modified the workshop for improved future delivery based on workshop exit survey feedback (Dataset III) and our observations. This was followed by the all-teaching staff survey (Dataset I) and staff interviews (Dataset II) which gathered staff perceptions on all FD, including our H5P workshops. For more detail regarding specific aspects of implementation, as they align with the above theoretical underpinnings, see *Extended data* ( Singleton *et al*., 2023)).

### FD implementation

**Figure 3.**
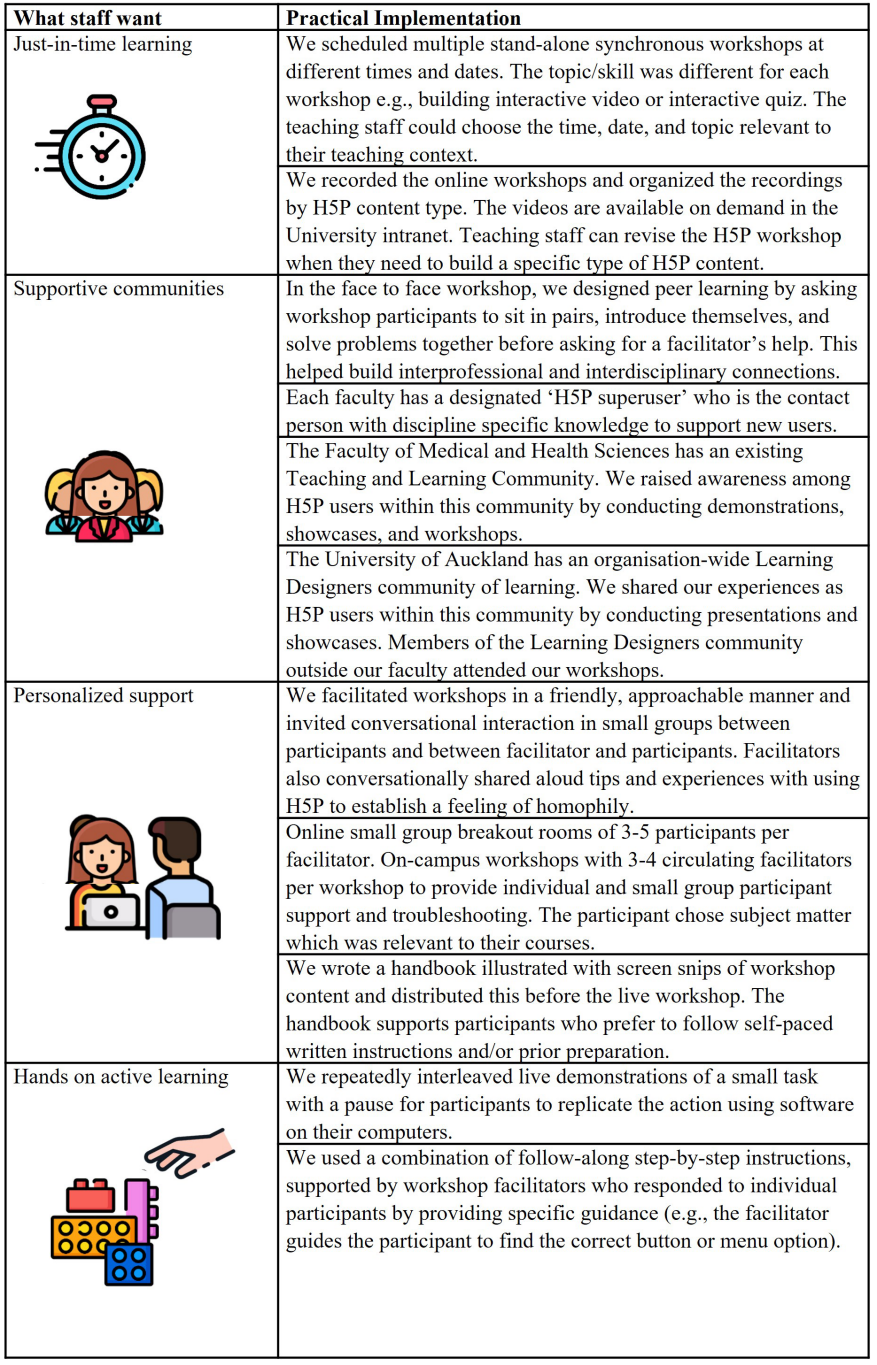
Implementing what teaching staff want in our faculty development workshop design. Icons in this figure are from Flaticon ( https://www.flaticon.com/). Author AC has a current paid premium annual subscription to Flaticon, which allows use of figures without further attribution. There is a free version of Flaticon, but users need to attribute the source.

In our study, we used opt-in workshops to develop staff capability for creating and embedding interactive online learning resources with H5P software (see *Extended data* ( Singleton *et al*., 2023). Our first workshop was held on campus in a computer lab. We held subsequent workshops via Zoom due to COVID-19 pandemic restrictions. Staff were invited to register for the workshop via the FMHS teaching and learning community distribution email list. The link to the Zoom workshop was then sent to those who registered. We held six 90-minute workshops, each with four to five facilitators and 25–40 learners. For details on how we designed our FD workshops to address what staff want, see Figure 3.

## Results

The all-teaching staff survey exposed limited staff capability for creating and managing online technology and pedagogy and highlighted the need for further FD.

### All-teaching staff survey (Dataset I)

**Figure 4.**
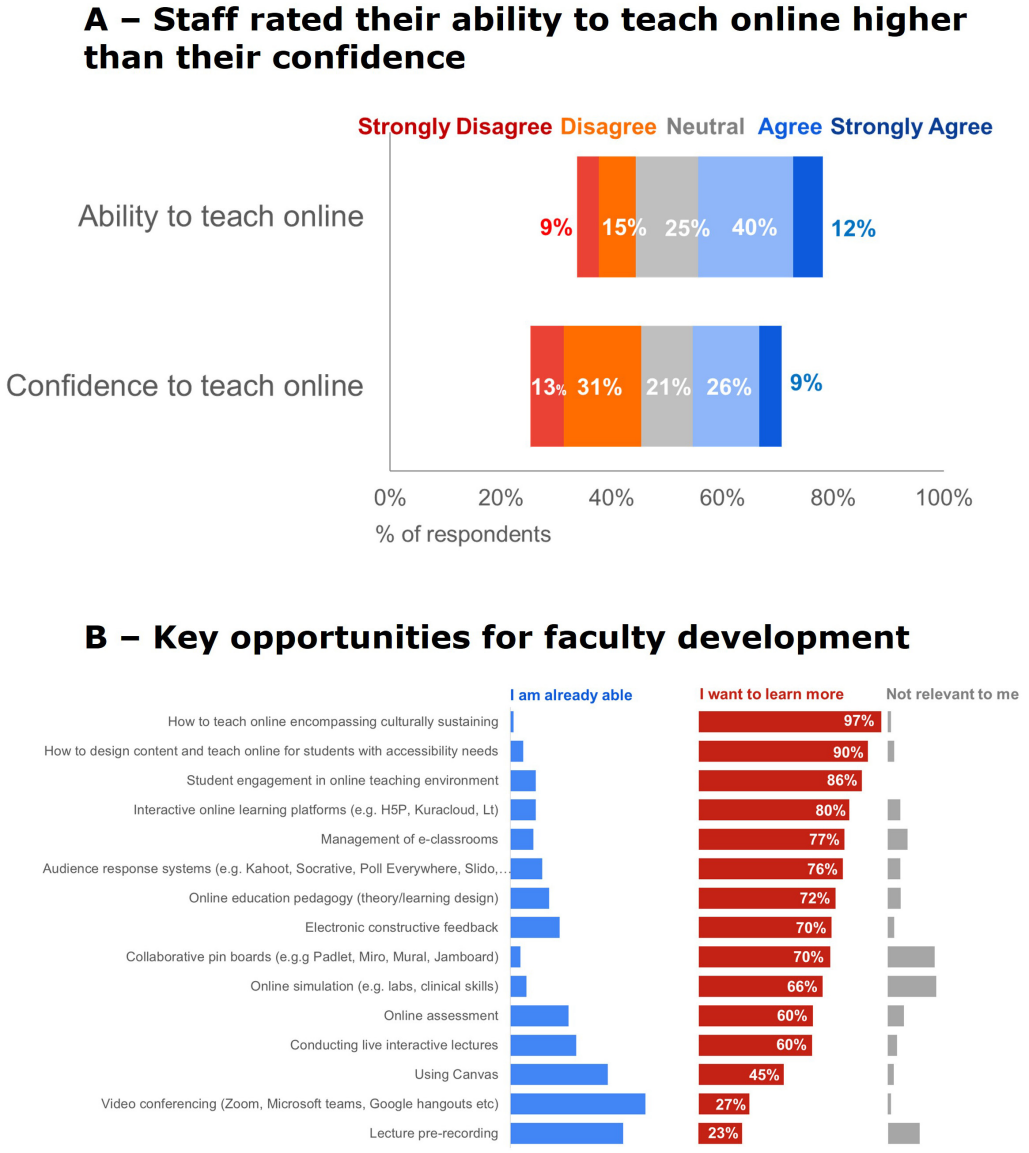
Ranked support for strengthening online teaching capability. **A** -Staff rated their ability to teach online higher than their confidence to teach online (79% response rate). **B** - Several key opportunities for FD were identified (77% response rate).

Eighty-six (19.1%) staff responded to the all-teaching staff survey to share their experiences and perceptions of online teaching. Three quarters (76%) of the respondents identified as female, this is reflective of our FMHS teaching staff population. In response to gender, one respondent answered ‘I don’t wish to answer’. Six respondents did not answer this question. Most of those surveyed had less than ten years of working in a teaching role. The largest age bracket (26%) for both male and female teaching staff was 40–59 years old. Forty-six respondents (56%) identified as New Zealand European. One respondent (3%) identified as Māori. Staff rated their ability to teach online higher than their confidence to teach online, where 52% of respondents generally agreed that they are able to teach online, but only 35% agreed that they were confident to do so. Staff ranked opportunities to strengthen their online teaching. The top four opportunities were: incorporating culturally sustaining pedagogies, accessibility, student engagement, and interactive learning platforms, Figure 4B.

**Figure 5.**
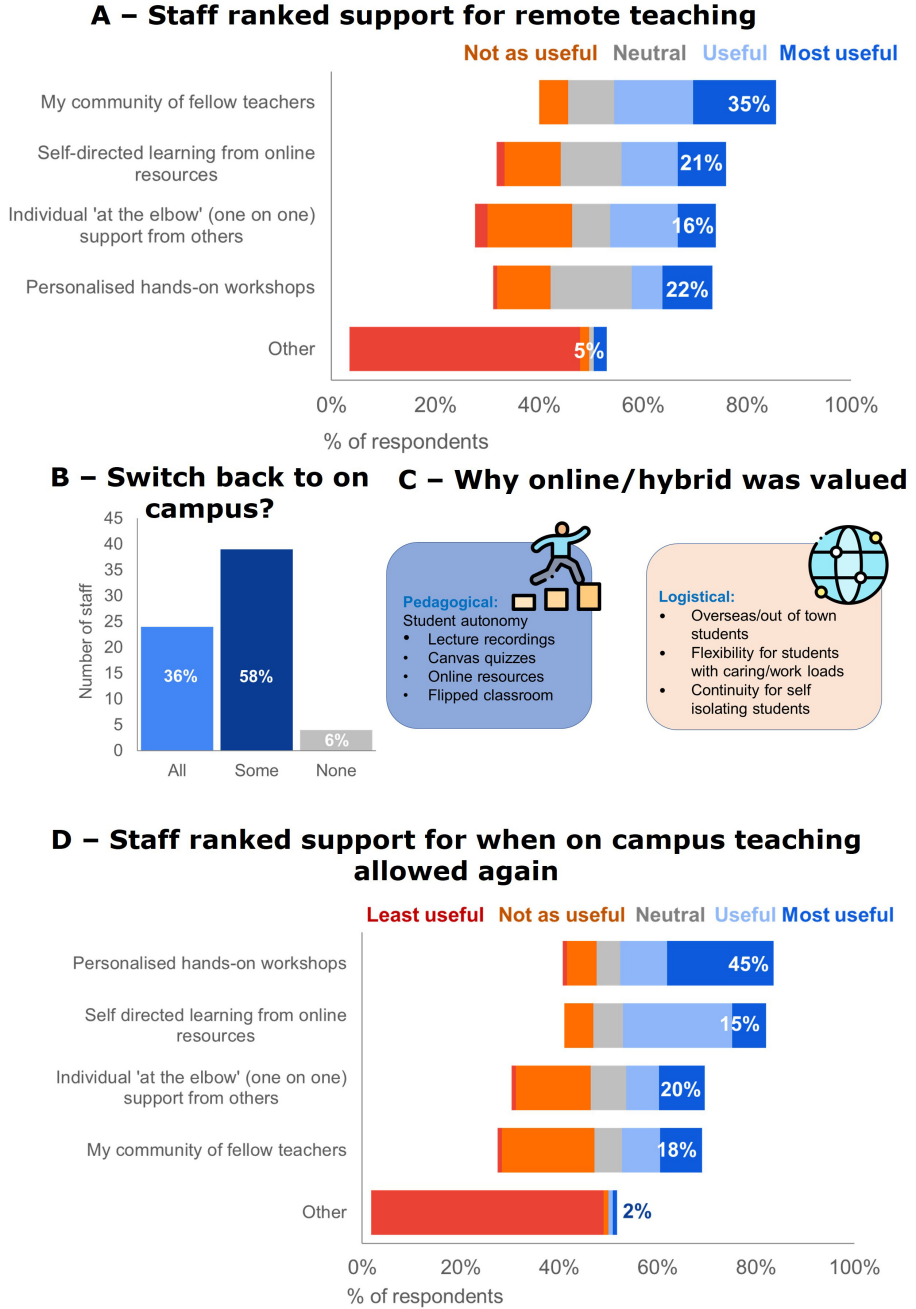
Staff ability and confidence and FD needs. **A** - Staff ranked their community of fellow teachers as most useful for strengthening online teaching capability during remote teaching (66% response rate). **B** - When on-campus teaching was allowed again some teaching staff switched all their teaching back, but a lot continued to teach online (78% response rate). **C** - A continuation of online/hybrid teaching was perceived as valuable for pedagogical and logistical reasons. Icons in this figure are from Flaticon ( https://www.flaticon.com/). **D** - Staff ranked faculty-wide workshops or seminars as most useful for strengthening online teaching capability post-pandemic (64 % response rate).

Teachers ranked their community of fellow teachers as the most useful for strengthening their online teaching capabilities, Figure 5A. When on-campus teaching was allowed, 58% of respondents would continue some online teaching (see Figure 5B), demonstrating a behavior change at BEME evaluation level three ( Steinert *et al.*, 2006) (see Figure 2). Reasons for this are both pedagogical (e.g., to allow for increased student autonomy, provision of resources for flipped classrooms) and logistical (e.g., for out-of-town students, flexibility for students, continuity for isolating students), Figure 5C. Staff ranked faculty-wide workshops or seminars as the most useful FD strategies they want for strengthening online teaching capability post-pandemic, Figure 5D.

### Teaching staff interviews (Dataset II)

Twenty staff participated in interviews. Seventeen participants were employed in teaching roles, and three held administrative or technical positions. Four participants were external to the University of Auckland and held clinical roles, teaching only occasionally.

Participants’ experience ranged from those relatively new to teaching to staff with several decades of experience. This pattern is reflected in the age range of the participants. Seven participants (35%) were aged 40–49 years. The participants identified with a range of ethnicities: New Zealand European (n = 7), European (n = 3), Indian (n= 2), British (n = 2), European American (n = 1), Fijian Indian (n = 1), and Chinese (n = 1), three participants did not state their identified ethnicity. Overall, staff acknowledged that effective online teaching requires different skills and techniques compared to on-campus teaching. Three major themes were developed from the data: 1) Time and timing, 2) Levels of support, and 3) Personalized support.

***Time and timing.*** The time required to develop online teaching strategies was seen as a burden on the workload and the mental and emotional load of staff. Therefore, prioritizing time was a significant consideration, which meant that many participants saw the appropriate timing of support and training as crucial. ‘Definitely we are all under a lot of pressure … so we don’t have time to just go through … every detail about what this platform does … and so oh I could implement this for this thing and then other software for other things *.*’ [Participant 16]

Many interviewees referred to feeling overloaded or that they were ‘drowning’. Not having the ‘bandwidth’ required was a common phrase used by participants, with several describing the situation as ‘mentally taxing’ or ‘overwhelming’ *.* A lack of educational software availability was not a theme identified in staff data; however, how to use the software and the time needed to integrate the use of software for teaching were key concerns.

***Levels of support.*** Interviewees identified the need for support from multiple levels of the UoA. This included policies at university management level to support good teaching practice, software use, and professional development. This support was particularly important for clinical teachers who were employed externally. ‘A bit more acknowledgment that actually we should be pretty good [at teaching], but we should also have the support and encouragement to upskill, time put aside to be able to do that *.*’ [Participant 9]

As an example of good institutional support, a site-wide license for H5P was rolled out in 2021. The number of users of H5P increased from less than 100 staff authors at the end of 2021 to over 400 staff authors and 11000 learners in semester one, 2022. This change across the institution demonstrates BEME evaluation level 4A outcome ( Steinert *et al.*, 2006) (see Figure 2).

At the local level, staff valued support from their colleagues and Communities of Practice (CoP). Colleagues were sources of inspiration, technical knowledge, and emotional support. Being part of a community of learners was seen as having several additional benefits. There was a perception from some that ‘a burden shared is a burden halved’ and the opportunity to get feedback and ask ‘stupid questions’ was seen as valuable.

***Personalized support.*** Regardless of whether support was provided in formal or informal formats, staff emphasized the importance of personalized support tailored to the specific stage of their learning journey and learning preferences. These included the opportunity to ask questions on an as-needed basis, being given advice on which tools might be best for specific purposes, seeing good practice in action, and developing pedagogical skills. ‘I think that would definitely be something, much more tailored to our needs rather than like a list of generic things that you can use. Just because I can doesn’t mean I need to and it might not be even suitable.’ [Participant 16]. ‘You don’t realize what you can do with something until you see somebody else actually do it and that’s been quite useful.’ [Participant 5]

### Teaching staff evaluation of FD workshops to use H5P software (Dataset III)

**Figure 6.**
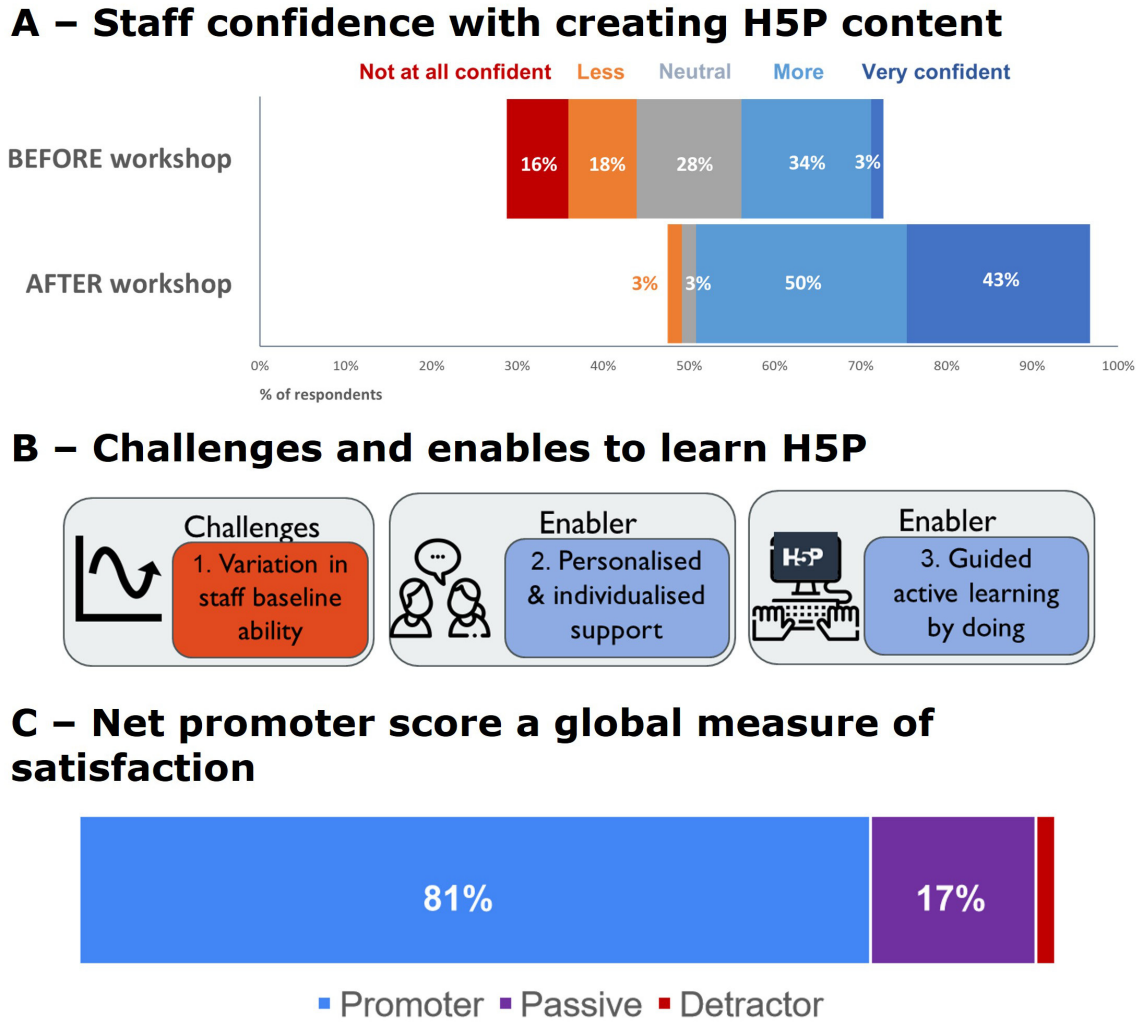
Staff ability and confidence to teach online with H5P. **A** - Confidence in teaching staff’s ability to create H5P resources increased from 37% being confident before the FD workshop to 93% after the workshop. **B** - Qualitative analysis of staff open-ended answers to workshop exit survey questions revealed challenges and enablers to learn how to use the H5P tool to create online content. Icons in this figure are from Flaticon ( https://www.flaticon.com/). **C** - Net Promoter Score. Most staff who responded to the exit survey would recommend this H5P workshop to a UoA friend or colleague interested in or needing to make active online learning content (59% response rate).

We thoughtfully designed FD workshops to respond to what staff want. In this example we designed a series of workshops to train staff to use H5P software. A total of 105 teaching staff attended the H5P workshops, and 59% of attendees responded to our exit survey. We did not collect demographic data. An overwhelming majority of staff (81% Net Promoter Score) would recommend our H5P workshop to a UoA friend or colleague interested in or needing to make interactive online learning resources (see Figure 6C). This is a BEME evaluation level 1 outcome, see Figure 2.

The FD workshops changed staff attitudes; confidence in our teaching staff’s ability to create H5P resources increased from 37% being confident before the FD workshop to 93% after the workshop, Figure 6A. This is a BEME evaluation level 2A ( Steinert *et al.*, 2006) outcome, Figure 2. As workshop facilitators, we found the variable baseline technical ability of staff challenging to ensure we met everyone’s FD requirements. However, staff reported they valued our workshop design which explicitly provided personalized support in small groups and active learning by doing (see Figure 6B).

From our multiple data set analysis, we illustrate positive educational outcomes at all BEME evaluation levels (see Figure 2).

### Student evaluation of teaching and interactive software (Datasets IV and V)

Student learning improved after providing engaging and interactive online resources, including assignments designed in H5P. Mean assignment grade increased significantly ( *p* <.001) between 2018 and 2019 from A-(83%) to A+(92%) (see *Extended data* ( Singleton *et al*., 2023)). ‘I found the online review articles to be intellectually stimulating, in that learning how to read a review article critically was a good skill to develop in this course. I will definitely use the techniques I learned from it in the future *.*’ [MEDSCI student, 2019]

Undergraduate MEDSCI 203 students (2018–2021) universally reported a preference for interactive approaches to online learning ( *n*=809 completed evaluation surveys, 48% average response rate), see *Extended data* ( Singleton *et al*., 2023). ‘Some more engaging steps involved in online lectures. Following the interactive route would help me, as it replaces the interaction usually present in normal lectures.’ [MEDSCI student, 2020]. ‘Record lectures that were produced to work in a way that is designed for online learning using visual gestures and diagrams to explain ideas instead of relying on videos that treat the online lectures as a neglected by–product of the past.’ [MEDSCI student, 2020] 

The trend for increased student performance following the provision of interactive online resources (interactive lecture videos and interactive online labs created in H5P) continued during compulsory online teaching delivery; 2018 mean course grade=B-, 2019 mean course grade=B, 2020 mean course grade=B+. These interactive online H5P resources continue to support hybrid/blended course delivery models now that on campus teaching has resumed. Online resources further enhance student learning (2021 mean course grade=B) by extending the learning environment and ensuring our taught content is more inclusive—by ensuring accessibility to students outside the classroom and reducing barriers to learning related to distance, time, and preference. ‘The [interactive] online review articles and lab assessments also reinforced the concepts taught in lectures. It was helpful in engaging with content in an enjoyable and practical way.’ [MEDSCI student, 2020]. ‘[The] online work allowed me to work at my own pace. The virtual labs were really well done.’ [MEDSCI student, 2021]. These findings demonstrate BEME evaluation level 4B outcome; by demonstrating improvement in student grades as a result of the educational intervention (see Figure 2).

### Triangulation – the overarching narrative

From the teaching staff, we identified time pressure and increased workload as challenges to creating interactive online teaching resources. Using a design thinking approach we delivered and evaluated workshops for a specific software (H5P) and this addressed what staff said they wanted to increase online teaching capability as identified by the all-teaching staff survey (Dataset I) and staff interviews (Dataset II). This software helped improve student learning by improving the ability of teaching staff to design and deliver interactive online content (Datasets IV and V). A select group of staff indicated they wanted to increase their online teaching capability,so we ran H5P workshops to meet that need. Then the all-staff survey, which included more teaching staff, showed that they too wanted the kind of skill building that we were offering through the workshops and suggested future FD opportunities. We show that when staff wants are met ( Figure 3) in FD that are underpinned by educational theory (see *Extended data* ( Singleton *et al*., 2023), then improvement in staff ability for online teaching can result in change across the institution ( Figure 2). Opportunities identified for future FD were how to implement universal accessibility in the design of online learning resources and how to teach online to build relationships and engagement, Figure 4B. Staff need protected time to increase their skills and develop learning resources.

## Discussion

Redecker *et al*. (2011) predicted that effective professional development would be personalized, motivating, engaging, efficient (including timely), and relevant. Eleven years later, our study confirms these predictions hold true in the online learning environment. We found that teachers’ interactive learning strategies and resources created during compulsory online teaching delivery supported and encouraged learners to engage. Not only this but student grades improved. Part of this grade improvement is likely because the software scaffolds teachers to provide effective feedback and to incorporate constructive alignment into feedback to students; actions that teachers may otherwise overlook without having such scaffolding to remind them. This finding also supports the notion that intrinsic motivation and interactive online learning are key to the needs of both teachers and learners as we rush towards an increasing proportion of tertiary education delivered online. We found teaching staff do not want more tools, technologies, or software. Instead, staff value FD for strengthening their online teaching capability in both technical and pedagogical aspects. Staff want to use existing technologies better. These findings are relevant to tertiary institutions wanting to sustainably engage staff to strengthen their online teaching capability.

For institutions offering FD initiatives for online teaching that are responsive to the wants of their teaching staff, we offer four recommendations (see Figure 3):

1. Provide just-in-time learning and allocate time for FD and staff to create online teaching material.
2. Foster supportive communities ( Buckley *et al.*, 2019; Lave & Wenger, 1991; Littlejohn, 2022; Yarris *et al.*, 2019)
3. Offer personalized support ( Downing & Dyment, 2013)
4. Design hands on active learning ( Steinert *et al.*, 2006).

While our recommendations are based on data from a medicine and health sciences context, they are unlikely specific to these disciplines. Instead, they resonate with broader education literature. Supportive communities, for example, speaks to the continuing importance of what Lave and Wenger (1991) first described as a CoP. CoPs–and the more recent Virtual CoPs (VcoPs) are understood as instrumental and intentional social tools for designing and developing learning environments ( Littlejohn, 2022; Yarris *et al.*, 2019).

However FD initiatives are provided, it is essential to design for active learning ( Freeman *et al.*, 2014) through fostering generative processing as described by Mayer (2017). Doing so supports teacher intrinsic motivation: autonomy, competence, and relatedness ( Deci & Ryan, 1985). Our research shows that time allocated for FD, just-in-time learning, personalized support, and supportive communities responds to what teachers want and keeps them motivated. These recommendations underpin strategies we used and can guide the upskilling and upscaling of FD.

### Strengths and limitations

We report a significant body of triangulated data to demonstrate FD efficacy at all BEME evaluation levels ( Steinert *et al.*, 2006). The qualitative component of our study privileges the voices and perspectives of teaching staff. Our findings contribute to an international conversation about how teachers want to be supported during the rapid and massive global transitions to online teaching. The focus of this study was on evaluating FD initiatives from the staff perspective. A related but secondary focus is on student preference for interactive approaches to online learning, (retrospective analysis of student evaluation data from 2018–2021) and was affected by several contextual factors during the pandemic which could have affected this outcome but were not controlled by the authors. However, the data reported for the observed improvement in students` performance following the introduction of interactive online content (H5P) was collected from pre-pandemic student cohorts (2018–2019). We do not know the extent to which the findings can be generalized according to demographic facets such as gender and ethnicity because we did not seek this information for datasets III, IV and V.. All our raw data has been made available, (see *Underlying data* ( Singleton *et al.*, 2023). We collected demographic data for the all-teaching staff survey (Dataset I) and staff interviews (Dataset II). These datasets could be used for future disaggregated analysis by gender and ethnicity. We would recommend to future researchers to gather such information where possible, to enable analysis of the bearing they have on learning and faculty development. We conducted this study in a single large faculty of New Zealand's leading university; not all findings may be generalizable to other institutions. The survey response rate was high enough to reach powered validity but still modest. This likely reflects the timing of the survey distribution during a pandemic, when staff were overwhelmed, juggling high workloads, and trying to rapidly adapt to online teaching, as reflected in their survey responses. Inferences made in the article about the impact of the FD interventions may similarly reflect the timing of the dataset I and II collection when staff were overwhelmed by workload and adapting to changes in general. Our next steps are to engage with staff in other disciplines and tertiary institutions post-pandemic to translate what we have learned into developing workshops to help guide other teaching staff on how to best support their peers locally.

## Conclusion

Globally, tertiary education is speedily ascending the diffusion of innovation curve for online teaching. Teachers urgently need support and increased capability. FD to strengthen online teaching capabilities is needed and welcomed by staff. To make FD opportunities effective, staff need to be allocated time and given appropriate support at all levels from the institution. Effective support can be found in CoPs and VCoPs, with expert help that is just-in-time and personalized to address what teachers want and their learning preferences. Responding to what teachers want enabled diffusion of innovation in online teaching capability across the institution and increased student learning. FD initiatives that align with what staff want are key to staff engagement. Here we have provided a guide for effective design and delivery of FD initiatives. We recommend those involved in FD respond to what staff want and underpin their FD with educational theory and practical workshop design.

## Data Availability

Figshare: Faculty development for strengthening online teaching capability: a mixed-method study of what staff want, evaluated with Kirkpatrick’s model of teaching effectiveness https://doi.org/10.17608/k6.auckland.c.6673781.v4 ( Singleton *et al*., 2023) This project contains the following underlying data: Dataset I_All teaching staff survey of opportunities to strengthen online teaching capabilities_v1 01-06-2023.csv (DATASET1.6.2023, 11:23184.95 kB) Dataset II_Interview question guide_v1 01-02-2023.docx (WORKFLOW1.6.2023, 13:3814.42 kB) Dataset II_Nvivo database on staff interview transcripts about faculty faculty development for online teaching_v1 01-06-2023.nvp (DATASET1.6.2023, 11:2315.44 MB) Dataset II_NVivo codebook of staff interview transcripts about faculty development for online teaching_v1 01-06-2023.docx (REPORT1.6.2023, 11:2319.89 kB) Dataset III_H5P workshop staff exit survey_v1 0-06-2023.csv (DATASET1.6.2023, 11:2333.95 kB) Dataset IVa_SET and OLE Course and Teacher Evaluation Reports MEDSCI 203_2018-2021_v1 01-06-2023.pdf (DATASET1.6.2023, 11:233.51 MB) Dataset IVb_Open ended student comments_teaching evaluations 2018_v1 01-06-2023.csv (DATASET1.6.2023, 11:2321.61 kB) Dataset IVb_Open ended student comments_teaching evaluations 2019 _v1 01-06-2023.csv (DATASET1.6.2023, 11:2328.49 kB) Dataset IVb_Open ended student comments_teaching evaluations 2021_v1 01-06-2023.csv (DATASET1.6.2023, 11:2324.52 kB) Dataset V_Raw student H5P assignment grades 2018-19_v1 01-06-2023.csv (DATASET1.6.2023, 11:2328.11 kB) Figshare: Faculty development for strengthening online teaching capability: a mixed-method study of what staff want, evaluated with Kirkpatrick’s model of teaching effectiveness https://doi.org/10.17608/k6.auckland.c.6673781.v4 ( Singleton *et al*., 2023) Electronic supplementary material_A. Table BEME (2006) modification of Kirkpatrick's levels of evaluation_21-06-2023.docx (PREPRINT1.6.2023, 11:4657.5 kB) Electronic supplementary material_B. All Teaching Staff Survey_21-06-2023 (PREPRINT21.6.2023, 11:5363.06 kB) Electronic supplementary material_C. All Teaching Staff Interview Questions_21-06-2023 (PREPRINT21.6.2023, 12:0557.24 kB) Electronic supplementary material_D. H5P Workshop Exit Survey_21-06-2023 (PREPRINT21.6.2023, 12:1056.9 kB) Electronic supplementary material_E. Faculty Development Workshop Design linked to theory_21-06-2023 (PREPRINT21.6.2023, 12:1462.03 kB) Electronic supplementary material_F. Figure 1 Example of a technology enhanced learning activity created with H5P Software_21-06-2023 (PREPRINT21.6.2023, 12:18552.15 kB) Electronic Supplementary Material_G. FD Workshop Design Plan_21-06-23 (PREPRINT21.6.2023, 12:47136.03 kB) Data are available under the terms of the Attribution-Non Commercial 4.0 International (CC BY-NC 4.0) license.

## References

[ref-1] Ando H Cousins R Young C : Achieving Saturation in Thematic Analysis: Development and Refinement of a Codebook. *Comprehensive Psychology.* 2014;3: 03.CP.3.4.

[ref-2] Blumenstein M : Synergies of Learning Analytics and Learning Design: A Systematic Review of Student Outcomes. *Learning Analytics.* 2020;7(3):13–32. 10.18608/jla.2020.73.3

[ref-3] Braun V Clarke V : Thematic analysis.In: *APA handbook of research methods in psychology, Vol 2: Research designs: Quantitative, qualitative, neuropsychological, and biological.*Washington, DC US: American Psychological Association;2012;2:57–71. 10.1037/13620-004

[ref-4] Brookfield S : Critically reflective practice. *J Con Educ Health.* 1998;18(4):197–205. 10.1002/chp.1340180402

[ref-5] Brown T Katz B : Change by Design. *J Prod Innov Manage.* 2011;28(3):381–383. 10.1111/j.1540-5885.2011.00806.x

[ref-6] Buckley H Steinert Y Regehr G : When I say … community of practice. *Med Educ.* 2019;53(8):763–765. 10.1111/medu.13823 30859612

[ref-7] Daniel M Gordon M Patricio M : An update on developments in medical education in response to the COVID-19 pandemic: A BEME scoping review: BEME Guide No. 64. *Med Teach.* 2021;43(3):253–271. 10.1080/0142159X.2020.1864310 33496628

[ref-8] Deci EL Ryan RM : Intrinsic Motivation and Self-Determination in Human Behavior.Boston, MA: Springer US;1985; [accessed 2022 Mar 2]. 10.1007/978-1-4899-2271-7

[ref-9] Downing JJ Dyment JE : Teacher Educators’ Readiness, Preparation, and Perceptions of Preparing Preservice Teachers in a Fully Online Environment: An Exploratory Study. *The Teacher Educator.* 2013;48(2):96–109. 10.1080/08878730.2012.760023

[ref-10] Freeman S Eddy SL McDonough M : Active learning increases student performance in science, engineering, and mathematics. *Proc Natl Acad Sci U S A.* 2014;111(23):8410–5. 10.1073/pnas.1319030111 24821756 PMC4060654

[ref-11] Jowsey T Deng C Weller J : General-purpose thematic analysis: a useful qualitative method for anaesthesia research. *BJA Educ.* 2021;21(12):472–478. 10.1016/j.bjae.2021.07.006 34840819 PMC8606608

[ref-12] Jowsey T Foster G Cooper-Ioelu P : Blended learning via distance in pre-registration nursing education: A scoping review. *Nurse Educ Pract.* 2020;44: 102775. 10.1016/j.nepr.2020.102775 32247200 PMC7195119

[ref-13] Kaminski J : Diffusion of innovation theory. *Canadian Journal of Nursing Informatics.* 2011;6(2):1–6. Reference Source

[ref-14] Lave J Wenger E : Situated learning: legitimate peripheral participation.Cambridge England; New York: Cambridge University Press,1991. 10.1017/CBO9780511815355

[ref-15] Littlejohn A : Transforming educators’ practice: how university educators learned to teach online from home during the Covid-19 pandemic. *High Educ Res Dev.* 2022;42(2):366–381. 10.1080/07294360.2022.2073982

[ref-16] Marder B Houghton D Erz A : Smile(y) - and your students will smile with you? the effects of emoticons on impressions, evaluations, and behaviour in staff-to-student communication. *Stud High Educ.* 2020;45(11):2274–2286. 10.1080/03075079.2019.1602760

[ref-17] Mayer RE : Using multimedia for e-learning. *J Comput Assist Learn.* 2017;33(5):403–423. 10.1111/jcal.12197

[ref-18] Plattner H Meinel C Leifer L : Design thinking: understand-improve-apply.[place unknown]: Springer Science & Business Media,2010. Reference Source

[ref-19] Redecker C Leis M Leendertse M : The future of learning: New ways to learn new skills for future jobs.Technical Note JRC60869, JRC-IPTS, Seville,2010. Reference Source

[ref-20] Redecker C Leis M Leendertse M : The Future of Learning: Preparing for Change.[place unknown].2011. Reference Source

[ref-21] Redmond P Heffernan A Abawi L : An Online Engagement Framework for Higher Education. *OLJ.* 2018;22(1):183–204. 10.24059/olj.v22i1.1175

[ref-22] Reichheld FF : The One Number You Need to Grow. *Harv Bus Rev.* 2003;81(12):46–54. 14712543

[ref-23] Rogers EM Singhal A Quinlan MM : Diffusion of innovations.In: *An integrated approach to communication theory and research.*[place unknown]: Routledge;2014;432–448.

[ref-30] Singleton R Ruiz Cosignani D Kam M : Faculty development for strengthening online teaching capability: a mixed-methods study of what staff want, evaluated with Kirkpatrick’s model of teaching effectiveness.The University of Auckland. Collection,2023. 10.17608/k6.auckland.c.6673781.v4 PMC10739185 38144874

[ref-24] Steinert Y Mann K Anderson B : A systematic review of faculty development initiatives designed to enhance teaching effectiveness: A 10-year update: BEME Guide No. 40. *Med Teach.* 2016;38(8):769–86. 10.1080/0142159X.2016.1181851 27420193

[ref-25] Steinert Y Mann K Centeno A : A systematic review of faculty development initiatives designed to improve teaching effectiveness in medical education: BEME Guide No. 8. *Med Teach.* 2006;28(6):497–526. 10.1080/01421590600902976 17074699

[ref-26] Yarris LM Chan TM Gottlieb M : Finding Your People in the Digital Age: Virtual Communities of Practice to Promote Education Scholarship. *J Grad Med Educ.* 2019;11(1):1–5. 10.4300/JGME-D-18-01093.1 30805087 PMC6375332

