# Supplementary material for: Faculty development for strengthening online teaching capability: a mixed-methods study of what staff want, evaluated with Kirkpatrick’s model of teaching effectiveness
Source: MedEdPublish (2016). 2023 Nov 30;13:127. Originally published 2023 Sep 12. [Version 2] doi: 10.12688/mep.19692.2 (PMC10739185; doi:10.12688/mep.19692.2)
Supplement: Peer review history

# Reviewer report: Reviewer response for version 2

**DOI:** 10.21956/mep.21480.r35442

**Published:** 2023-12-21

**Authors:** Tom Olney

**Affiliations:** 1 The Open University, Milton Keynes, England, UK

The addition of part B to Figure 1 is welcome, and clarifies the order of data collection which was caused by the numbering of the data sets in a way that prioritized the student data. Thank you for adding this element to the paper and good luck.

Have any limitations of the research been acknowledged?

Yes

Is the work clearly and accurately presented and does it cite the current literature?

Yes

If applicable, is the statistical analysis and its interpretation appropriate?

Yes

Are all the source data underlying the results available to ensure full reproducibility?

Yes

Is the study design appropriate and is the work technically sound?

Yes

Are the conclusions drawn adequately supported by the results?

Partly

Are sufficient details of methods and analysis provided to allow replication by others?

Yes

Reviewer Expertise:

Online and distance learning, learning design, professional development, training

I confirm that I have read this submission and believe that I have an appropriate level of expertise to confirm that it is of an acceptable scientific standard.

# Reviewer report: Reviewer response for version 2

**DOI:** 10.21956/mep.21480.r35443

**Published:** 2023-11-30

**Authors:** Barbara Jennings

**Affiliations:** 1 Norwich Medical School, Faculty of Medicine and Health, University of East Anglia, Norwich, England, UK

Thank you for the revised version of the manuscript. You have addressed all of my queries and I agree with reviewer 2 that your paper will be valuable to the MEP readership and clinical education community. Thank you for sharing your work.

Have any limitations of the research been acknowledged?

Yes

Is the work clearly and accurately presented and does it cite the current literature?

Yes

If applicable, is the statistical analysis and its interpretation appropriate?

Yes

Are all the source data underlying the results available to ensure full reproducibility?

Yes

Is the study design appropriate and is the work technically sound?

Yes

Are the conclusions drawn adequately supported by the results?

Partly

Are sufficient details of methods and analysis provided to allow replication by others?

Yes

Reviewer Expertise:

Genetics education; faculty development; technology enhanced learning

I confirm that I have read this submission and believe that I have an appropriate level of expertise to confirm that it is of an acceptable scientific standard.

# Reviewer report: Reviewer response for version 1

**DOI:** 10.21956/mep.21093.r35083

**Published:** 2023-11-24

**Authors:** Tom Olney

**Affiliations:** 1 The Open University, Milton Keynes, England, UK

Thank you for asking me to review this paper which describes a series of workshops designed to support staff at UoA in designing and developing online material during and after the COVID pandemic when face to face teaching was not available. The authors have attempted to link 5 sets of survey data used around these workshops in order to evaluate their effectiveness at improving the online teaching capacity of the staff. In general the paper is well structured and well written, the arguments are logical and address a topical subject around the effectiveness of professional development in education which is always relevant but has perhaps been brought more sharply into focus by emergency online requirements. 

 I have access to the comments made by reviewer one, so I won't repeat the points made there which I agree with. Having said that I would like to develop the point that was made by reviewer 1 about the timelines associated with the collection, analysis and interpretation of the five datasets. I think this is something that does need addressing because I also found them quite unclear and difficult to follow. In particular I had some problems understanding the timing of the collection of Datasets I and II which are provided as the basis for 'what staff want'. Both figure 1 and 3 suggest that this data was collected before the workshops took place, and was then used to shape the pedagogy. However, in the paragraph describing Dataset I and II on page 4 it describes the aim of these instruments as being 'to capture a holistic view of the teaching staff's perceptions of all the FD available and how they have applied any knowledge or skills acquired from them.' which suggest the workshops had already happened. Some clarification here is needed. 

 Further, the results from Dataset I which are provided in figure 4 don't seem to really tally with the findings established in figure 3? Figure 4 shows a comparison of perceptions of confidence vs ability and some preferences for what staff want to learn about in online teaching, but it's not clear how these results have informed the pedagogical design of the workshop that is presented by figure 3. In my opinion establishing this link more closely needs some attention and would improve the quality of the paper. 

 Otherwise, thank you for sharing your work and, once published, I'm sure it will provide an important contribution to the literature.

Have any limitations of the research been acknowledged?

Yes

Is the work clearly and accurately presented and does it cite the current literature?

Yes

If applicable, is the statistical analysis and its interpretation appropriate?

Yes

Are all the source data underlying the results available to ensure full reproducibility?

Yes

Is the study design appropriate and is the work technically sound?

Yes

Are the conclusions drawn adequately supported by the results?

Partly

Are sufficient details of methods and analysis provided to allow replication by others?

Yes

Reviewer Expertise:

Online and distance learning, learning design, professional development, training

I confirm that I have read this submission and believe that I have an appropriate level of expertise to confirm that it is of an acceptable scientific standard, however I have significant reservations, as outlined above.

# Response

**Published:** 2023-11-27

**Authors:** Rachelle Singleton

**Affiliations:** School of Medical Sciences, The University of Auckland, Auckland, Auckland, New Zealand

Dear Tom Olney,

 I sincerely appreciate the time and effort you have invested in reviewing our paper submitted to MedEdPublish. Your detailed feedback has provided us with a roadmap for revisions to enhance the quality and clarity of our work. Here's a summary of the modifications made in response to your feedback:

 Methods:

 1. The methods section has been revised to incorporate a more in-depth explanation of the timelines associated with the collection, analysis, and interpretation of the five datasets. Fig 1. has been updated to include a part B) that visually complements the new text we have added under the ‘Datasets’ Methods sub-heading. Datasets I and II were prospective datasets collected after the H5P workshops and these were designed to capture a holistic view of teaching staff’s perceptions of *all* the FD available, including our H5P workshops (retrospective dataset III) and how they have applied any knowledge or skills acquired from them. We retain part A) of Fig 1. as originally submitted, to emphasise the dataset ordering within the paper which prioritises the staff perception of faculty development for teaching.

 2. We have included the month and year of data collection for each dataset under the sub-heading ‘Data collection: sampling and saturation.’

 3. We have added clarifying text for Dataset I (under ‘Data collection: sampling and saturation’) that this survey included teaching staff perceptions of *all* the FD available, *including our H5P workshops (Dataset III),* and how they have applied any knowledge or skills acquired from them.

 4. We have added clarifying text for Dataset III (under ‘Data collection: sampling and saturation’) to explain that *workshop exit surveys informed iterative design of subsequent workshops within a series of six workshops held across June - October 2021*.

 5. We have added clarifying text for the last paragraph (under ‘FD workshop design’) to ensure it is clearer to the reader that the all-teaching staff survey (Dataset I) and staff interviews (Dataset II) gathered staff perceptions on all FD, including our H5P workshops, after we delivered workshops that we believed would address what staff wanted. The H5P workshop exit surveys (Dataset III) shaped the pedagogical design of the workshops and the themes identified in the staff interviews (Dataset II) align with our implementation of what staff want in our faculty development workshop design (Fig 3.).

 Results:

 1. We have added clarifying text for the last paragraph (under ‘Triangulation – the overarching narrative’) to ensure it is clearer how the separate datasets have informed the pedagogical design of the workshop that is presented by Fig 3. That being that a select group of staff indicated they wanted to increase their online teaching capability and so we ran H5P workshops to meet that need. Then the all-staff survey, which included more teaching staff, showed that they too wanted the kind of skill building that we were offering through the workshops and suggested future FD opportunities.

 In addition to your insightful comments, we've also considered suggestions from the other reviewer. Collectively, these changes have significantly elevated the overall quality and impact of our paper. Again, thank you for your constructive feedback. I hope that our revisions meet your expectations and I look forward to hearing your thoughts on the modified paper.

# Reviewer report: Reviewer response for version 1

**DOI:** 10.21956/mep.21093.r35087

**Published:** 2023-10-24

**Authors:** Barbara Jennings

**Affiliations:** 1 Norwich Medical School, Faculty of Medicine and Health, University of East Anglia, Norwich, England, UK

I enjoyed reading this article describing an evaluation study, primarily exploring an organisation’s faculty development (FD) initiative to support online teaching. As a secondary aim the research team evaluated teaching effectiveness by surveying student perspectives and measuring learning gain before and after the intervention linked to the roll out of H5P Software (which is used to share and reuse HTML5 content and applications).

 The introductory paragraphs presented the rationale for the study by highlighting the problems encountered in higher education, when teaching had to pivot rapidly from campus to online – and the lack of FD research to inform interventions. The authors designed a study to address what staff need and want from a FD initiative to enhance online teaching capability, alongside the introduction of H5P software (which provides an effective tool for storing and presenting learning objects for asynchronous online lessons). They also used a modified Kirkpatrick model to evaluate teaching effectiveness after the FD initiatives had been incorporated.

 The methodology, research governance, datasets and analysis steps are all clearly described. An additional strength is the commitment to open-data-sharing and transparency with respect to the FD initiatives, including surveys; exemplar lessons; and the full datasets: they can all be viewed on Figshare, via the university site: https://doi.org/10.17608/k6.auckland.c.6673781.v4 ( Singleton *et al*., 2023)

 Furthermore, the authors describe efforts to reduce the risk of bias: the longitudinal FD initiative ran in parallel with mixed-methods research, carried out by authors who describe their risk of positive bias. Therefore, the team outsourced aspects of data collection and analysis to an independent consulting agency.

 Limitations: the study design and datasets collected are clearly illustrated in the helpful diagram in figure 1 – but the timelines for collection and analysis of each (and impact on interpretation) are less clear cut. For example, ethics approval was secured in November 2021 for the over-arching mixed methods study and dataset 5 from the undergraduate students was from 2018 to 2021. Also, inferences made in the article about the impact of the FD interventions and survey response rates (see discussion) may have reflected the timing of the project when staff were overwhelmed by workload and adapting to changes in general. Finally, the retrospective study design may have precluded the collection of full sets of demographic data – what do the authors think about these issues?

 The big themes that emerged about what teachers need will resonate with faculty development teams, and the infographic in Figure 3 is very helpful. The 4 main recommendations for just-in-time blended training, hands on active learning, communities of practice and personalised support with the help of “H5P superusers” merit being shared widely.

 A few minor manuscript/syntax problems were noted, e.g., mentioning Meyer principles but not having in-text citation in the Introduction.

 I think this article will be of interest to most clinical-school teachers who want to understand and apply principles of online teaching and instructional design; and their impact on student experience and learning. Our changing roles as educators are inherently demanding, and we need enjoyable and effective professional development activities to improve our confidence and skills – this paper describes some comprehensive protocols that others could follow. Also, the research findings and final conclusions will be of particular importance to faculty development leads and programme convenors - and have implications for resource allocation and the organisational leaders in clinical schools.

 Thank you to the research and educational team for sharing their important project and findings.

Have any limitations of the research been acknowledged?

Yes

Is the work clearly and accurately presented and does it cite the current literature?

Yes

If applicable, is the statistical analysis and its interpretation appropriate?

Yes

Are all the source data underlying the results available to ensure full reproducibility?

Yes

Is the study design appropriate and is the work technically sound?

Yes

Are the conclusions drawn adequately supported by the results?

Partly

Are sufficient details of methods and analysis provided to allow replication by others?

Yes

Reviewer Expertise:

Genetics education; faculty development; technology enhanced learning

I confirm that I have read this submission and believe that I have an appropriate level of expertise to confirm that it is of an acceptable scientific standard.

## References

[1] : Faculty development for strengthening online teaching capability: a mixed-methods study of what staff want, evaluated with Kirkpatrick’s model of teaching effectiveness. *MedEdPublish* .2023;13: 10.12688/mep.19692.1 10.12688/mep.19692.1 38144874 PMC10739185

# Response

**Published:** 2023-11-27

**Authors:** Rachelle Singleton

**Affiliations:** School of Medical Sciences, The University of Auckland, Auckland, Auckland, New Zealand

Dear Barbara Jennings,

 I sincerely appreciate the time and effort you have invested in reviewing our paper submitted to MedEdPublish. Your detailed feedback has provided us with a roadmap for revisions to enhance the quality and clarity of our work. We have made the timelines for data collection and analysis of each (and impact on interpretation), clearer in our revised paper. Here's a summary of the modifications made in response to your feedback:

 Introduction:

 1. We have included the in-text citation where we mention Meyer principles.

 Methods:

 1. The methods section has been revised to incorporate a more in-depth explanation of the timelines associated with the collection, analysis, and interpretation of the five datasets. Fig 1. has been updated to include a part B) that visually complements the new text we have added under the ‘Datasets’ Methods sub-heading. Datasets I and II were prospective datasets collected after the H5P workshops and these were designed to capture a holistic view of teaching staff’s perceptions of *all* the FD available, including our H5P workshops (retrospective dataset III) and how they have applied any knowledge or skills acquired from them. We retain part A) of Fig 1. as originally submitted, to emphasise the dataset ordering within the paper which prioritises the staff perception of faculty development for teaching.

 2. We have included the month and year of data collection for each dataset under the sub-heading ‘Data collection: sampling and saturation.’

 3. We have added clarifying text for Dataset I (under ‘Data collection: sampling and saturation’) that this survey included teaching staff perceptions of *all* the FD available, *including our H5P workshops (Dataset III),* and how they have applied any knowledge or skills acquired from them.

 4. We have added clarifying text for Dataset III (under ‘Data collection: sampling and saturation’) to explain that *workshop exit surveys informed iterative design of subsequent workshops within a series of six workshops held across June - October 2021*. 5. We have added clarifying text for the last paragraph (under ‘FD workshop design’) to ensure it is clearer to the reader that the all-teaching staff survey (Dataset I) and staff interviews (Dataset II) gathered staff perceptions on all FD, including our H5P workshops, after we delivered workshops that we believed would address what staff wanted. The H5P workshop exit surveys (Dataset III) shaped the pedagogical design of the workshops and the themes identified in the staff interviews (Dataset II) align with our implementation of what staff want in our faculty development workshop design (Fig 3.).

 Results:

 1. We have added clarifying text for the last paragraph (under ‘Triangulation – the overarching narrative’) to ensure it is clearer how the separate datasets have informed the pedagogical design of the workshop that is presented by Fig 3. That being that a select group of staff indicated they wanted to increase their online teaching capability and so we ran H5P workshops to meet that need. Then the all-staff survey, which included more teaching staff, showed that they too wanted the kind of skill building that we were offering through the workshops and suggested future FD opportunities.

 Strengths and limitations:

 1. We have added clarifying text to the ‘Strengths and limitations’ section to ensure that the reader is aware that we do not know the extent to which the findings can be generalized according to demographic facets such as gender and ethnicity because we did not seek this information for datasets III, IV and V, which were retrospective elements of the study design. We explain that all our raw data has been made available, (see Underlying data (Singleton et al., 2023) and that we collected demographic data for the all-teaching staff survey (Dataset I) and staff interviews (Dataset II). These datasets could be used for future disaggregated analysis by gender and ethnicity. We would recommend to future researchers to gather such information where possible, to enable analysis of the bearing they have on learning and faculty development.

 2. We already address in this section how survey response rates may have reflected the timing of the project when staff were overwhelmed by workload and adapting to changes in general. We have added to the ‘Strengths and limitations’ sections to indicate that the inferences we make in the article about the impact of the FD interventions may similarly reflect the timing of the dataset I and II collection, when staff were overwhelmed by workload and adapting to changes in general.

 In addition to your insightful comments, we've also considered suggestions from the other reviewer. Collectively, these changes have significantly elevated the overall quality and impact of our paper. Again, thank you for your constructive feedback. I hope that our revisions meet your expectations and I look forward to hearing your thoughts on the modified paper.
